# Cincinnati Academy of Medicine

**Published:** 1877-10

**Authors:** 


### PROCEEDINGS OF THE CINCINNATI ACADEMY OF MEDICINE.

---

*Case of Pyelitis.*—Dr. Kemper exhibited some pathological specimens, and said: These specimens are from a case of pyelitis of four years'standing. The only cause of the disease that I could discover is the uric acid diathesis. The symptoms of the disease took their rise almost imperceptibly. The urine was full of uric acid crystals during the four months I had charge of the case. There is no history of stone in the kidney or bladder. My judgment is that the uric acid crystals originated the suppurating nephritis in the left kidney. The inflammation extended to the ureter and bladder. The base of the bladder and a circle about the orifice of the left ureter are strongly marked by chronic inflammation. The left ureter is greatly hypertrophied, and its caliber diminished. The sigmoid flexure of the colon is fat and hypertrophied, and adherent to the ureter, and just behind this adhesion the ureter was adherent to the sheath of the psoas muscle. Just above this point is an irregularly-shaped sac, made out of the dilation of the ureter and sheath of the psoas muscle, the fibers of which are beginning to be infiltrated with pus. The pyramidal structure of the kidney is entirely destroyed, and the cortical portion nearly so. The right kidney and ureter are healthy, but enlarged from prolonged increased activity. At the autopsy the abdominal cavity was filled with pus, and the peritoneum and intestines softened by inflammation.

The course of the disease seems to have been this: The inflammation in the kidney extended to the ureter, and from that to the sigmoid flexure of the colon and the psoas muscle. The pressure of these two parts upon the ureter caused its diminished caliber to to be easily clogged by a uric acid crystal or a drop of pus. The clogging, repeated from time to time, created the sac. A residue of pus in the sac worked its way downwards and backwards into the sheath of the psoas muscle, forming a large abscess. A stoppage of the ureter caused the sac to fold so as to distend its walls. A brisk natural action of the bowels occurring under these circumstances caused a slight rupture of the frail adhesions between the ureter and sheath of the psoas muscle. Pus escaped into the abdominal cavity, and death was speedy and inevitable.

Dr. Longworth remarked, the general appearance of the specimen would seem to indicate tubercular disease of the kidney.

Dr. Kemper replied that there was no clinical history of tubercle in any other organ; and there never had been tubercular disease in the family. The right kidney was healthy. In tubercular pyelitis both kidneys are generally affected. Tubercle does not select the kidney by preference, and there is no clinical history of any determining cause that would satisfy us that tubercle had in this case departed from its invariable habit.

*A Case of Meningeal Rheumatism.*—Dr. McMechan reported the following case: On September 21st, 1877, Joseph Plaspohl, aged 14 years, was taken with a hard chill, which lasted about an hour. Fever followed the chill, and most intense pain in the right hip joint and in the head, accompanied the fever; vomiting took place quite frequently on the first day of the disease. On the second day of the attack I was called, and found the temperature 105°, the pulse 120, and the vomiting still continuing. The pupils were dilated; the patient was delirious most of the time, and the delirium was so intense at night, that he had to be held in bed; the bowels were constipated, and the pain in the head was quite severe, but not so intense as that in the hip joint. Salicylic acid and morphine were ordered for the patient internally, and ice-water was applied to the head. On the third day of the disease the vomiting ceased, but the pupils still remained dilated, and the pain in the head and in the hip joint was just as severe as at first. The delirium continued. The temperature was 103½°, and the pulse 130. Bromide of potassium in thirty-grain doses every four hours was given, but failed to control the delirium in the slightest degree. Drs. Harp and Kane were called in consultation. On the sixth day of the disease, and on the fifth day of the treatment, and until within three hours of expiring, he still complained of pain in the head, most excruciating pain in the right hip joint, and was still delirious. The other symptoms previously described also remained as severe as at the beginning of the treatment. The diagnosis of the case was meningeal rheumatism. Niemeyer, in his work on the Practice of Medicine, reports a case of this disease in the following words, viz: "I myself have only seen one case where the moderate increase of bodily temperature, the delirium in the first stage, and the subsequent coma, together with excessive retardation of the pulse and repeated vomiting, left no doubt as to the nature of the disease." He also says: "In a dissertation written by Dr. Flamm, under the direction of my colleague, Koehler, on 'Meningeal Symptoms in Acute Rheumatism,' cases are reported where *post-mortem* examination showed the presence of inflammatory disease of the meninges. The *Allgemeine Medizinische Central-Zeitung*, No. 58, 1877, contains a very interesting article on this subject. In said article cases occurring in the practice of some of the most distinguished medical writers are related. That such a disease as meningeal rheumatism exists cannot be doubted, as many well-authenticated cases of the disease have been reported.

